# Dataset of 16S ribosomal DNA sequence-based identification of endophytic bacteria isolated from healthy and diseased Sabah red algae, *Kappaphycus alvarezii*

**DOI:** 10.1016/j.dib.2023.109785

**Published:** 2023-11-07

**Authors:** Vernon Vest Mangun, Rajeena Sugumaran, Wilson Thau Lym Yong, Nur Athirah Yusof

**Affiliations:** Biotechnology Research Institute, Universiti Malaysia Sabah, Jalan UMS, Kota Kinabalu, Sabah 88400, Malaysia

**Keywords:** Macroalgae, Sabah seaweed, Endophytic bacteria, 16S rDNA, *Kappaphycus alvarezii*, Seaweed disease, Climate change

## Abstract

Bacterial endophytes play a vital role in the growth and fitness of host plants from infection by phytopathogens. To our knowledge, however, little information is available on the endophytic bacterial composition in healthy and diseased *Kappahycus alvarezii*, one of the most important major sources of carrageenan industries, especially in Sabah. The main idea was to analyze and compare the composition of endophytic bacterial communities in healthy and diseased *K. alvarezii* isolated from Sabah, Malaysia. The data reveals the composition of endophytic bacterial microbiomes in healthy and diseased *K. alvarezii* isolated from Sabah. The isolated endophytes were identified using 16S rDNA sequencing. Taxonomic identification and phylogenetic tree analysis were done using the online BLAST (blastn) and MEGA11 software, respectively. The data presents the diversity of bacterial endosphere microbiomes found in healthy *K. alvarezii* which are composed of *Bacillus, Cytobacillus* and *Priestia* whereas *Vibrio* and *Micrococcus* occurred exclusively in the diseased *K. alvarezii*. Microbial comparative analysis between the healthy and diseased seaweed points to the potential of several *Bacillus* strains that may have biocontrol potential against *Vibrio* infection in seaweed such as the ice-ice disease. Raw data files are available at the GenBank, NCBI database under the accession number MZ570560 to MZ570580.

Specifications Table

**Table:** 

Subject	Microbiology: Microbiome
Specific subject area	Analysis of endophytic microbiomes present in the healthy and infected seaweed of *K. alvarezii*
Type of data	Table and figure
How the data were acquired	Amplification and sequencing of bacterial 16S rDNA amplicons of endophytic bacterial isolates from *K. alvarezii*
Data format	Raw and Analysed
Description of data collection	Sterilized healthy and diseased *K. alvarezii* samples were cut into small pieces, vortexed vigorously and incubated in nutrient broth for 3 h to complete the release of endophytic microorganisms from the host tissue. The bacterial culture was plated on several media and incubated for 15 days at 28 °C. All isolates were preserved at −80 °C. DNA extraction and 16S rRNA gene amplicon sequencing were done. The 16S rDNA sequences were identified using the online BLAST (blastn). The MEGA11 software was used to construct a phylogenetic tree.
Data source location	• Institution: Biotechnology Research Institute, Universiti Malaysia Sabah • City/Town/Region: Sabah • Country: Malaysia
Data accessibility	• Repository name: GenBank database, NCBI • Data identification number: OQ552790-OQ552820 Direct URL to data: Accession numbers were provided in Tables 1 and 2. (https://www.ncbi.nlm.nih.gov/nuccore/?term=OQ552790%3AOQ552820%5Baccn%5D)

## 1 Value of the Data

• The data presents the diversity of bacterial endosphere microbiomes found in healthy and diseased *K. alvarezii* from Sabah using DNA sequencing analysis.
• The data show that the dominant endophytic bacterial genera were *Bacillus, Cytobacillus* and *Priestia* in healthy *K. alvarezii* whereas *Vibrio* and *Micrococcus* occurred exclusively in diseased *K. alvarezii*.
• The data provides important information on the presence of endophytic bacteria in healthy *K. alvarezii* which are potentially the key determinant of seaweed health and productivity.
• The data can serve as guidance for the selection and determination of potential endophytic microbes associated with biocontrol and plant-growth-promoting properties.
• The data is useful for the scientific committees to use endophytic microbiomes as potential biofertilizers, biopesticides, and biocontrol agents in seaweed farming.

## 2 Objective

The data was collected to access the bacterial endophyte communities in healthy *K. alvarezii* isolated from Sabah, Malaysia. Comparative analysis between the bacterial communities in the healthy and diseased *K. alvarezii* was done to identify endophytic bacteria that are exclusive in healthy *K. alvarezii*. The data serves as a platform for researchers to explore the potential of endophytic bacteria for plant growth promotion and biocontrol.

## 3 Data Description

### 3.1 Taxonomic Identification of Endophytes

The raw dataset contained 16S rDNA sequences of endophytic bacteria from healthy and diseased Sabah marine red algae, *Kappaphycus alvarezii.* This data was used to identify and investigate the endophytic microbiome in both healthy and diseased marine red algae, *K. alvarezii*. The taxonomic identification of the endophytic isolates was performed using the basic local alignment search tool (BLAST) (https://blast.ncbi.nlm.nih.gov/Blast.cgi). Tables 1 and 2 listed the outputs from the taxonomic identification of endophytic bacteria, which consisted of bacteria species, accession numbers of deposited sequences, accession numbers of the nearest matches, query cover, identities, gaps, and E-values from healthy and diseased *K. alvarezii*, respectively.

**Table 1:** Taxonomic identification of endophytic bacteria from healthy *K. alvarezii* using the NCBI Basic Local Alignment Search Tool (BLAST).

Bacteria ID	Bacteria species	Accession No. of deposited sequences	Accession No. of the nearest match	Query cover	Identities	Gaps	E-value
H-11	*Bacillus altitudinis*	OQ552790	OQ221514	100 %	100.00 %	0 %	0.0
H-115	*Bacillus altitudinis*	OQ552791	OQ295969	100 %	100 %	0 %	0.0
H-13	*Bacillus altitudinis*	OQ552792	OQ295976	100 %	100 %	0 %	0.0
H-13A	*Cytobacillus kochii*	OQ552793	MW435480	99 %	99.17 %	0 %	0.0
H-15B	*Bacillus sp.*	OQ552794	KX601097	99 %	99.58 %	0 %	0.0
H-1A	*Bacillus cereus*	OQ552795	MN746165	99 %	98.69 %	0 %	0.0
H-1C	*Bacillus altitudinis*	OQ552796	MW474842	99 %	99.45 %	0 %	0.0
H-21B	*Cytobacillus kochii*	OQ552797	MW435480	99 %	99.24 %	0 %	0.0
H-23	*Bacillus sp.*	OQ552798	MN932155	99 %	99.24 %	0 %	0.0
H-31	*Bacillus altitudinis*	OQ552799	KC441770	99 %	99.51 %	0 %	0.0
H-3A	*Bacillus cereus*	OQ552800	MT545090	100 %	98.19 %	0 %	0.0
H-3B	*Bacillus cereus*	OQ552801	MT332156	99 %	99.31 %	0 %	0.0
H-3C	*Bacillus cereus*	OQ552802	MK743993	99 %	99.52 %	0 %	0.0
H-41A	*Priestia megaterium*	OQ552803	MH762123	99 %	99.52 %	0 %	0.0
H-45	*Bacillus sp.*	OQ552804	MG470672	99 %	99.24 %	0 %	0.0

**Table 2:** Taxonomic identification of endophytic bacteria from diseased *K. alvarezii* using the NCBI Basic Local Alignment Search Tool (BLAST).

Bacteria ID	Bacteria species	Accession No. of deposited sequences	Accession No. of the nearest match	Query cover	Identities	Gaps	E-value
D-12	*Bacillus sp.*	OQ552805	MG470672	99 %	99.59 %	0 %	0.0
D-12A	*Micrococcus luteus*	OQ552806	AY512635	99 %	98.85 %	0 %	0.0
D-12C	*Bacillus sp.*	OQ552807	MN511801	99 %	95.39 %	0 %	0.0
D-13A	*Bacillus altitudinis*	OQ552808	OP893808	100 %	100 %	0 %	0.0
D-31B	*Bacillus sp.*	OQ552809	MG470672	99 %	98.19 %	0 %	0.0
D-31C	*Alkalihalobacillus sp.*	OQ552810	CP012475	99 %	83.02 %	0 %	0.0
D-32A	*Bacillus cereus*	OQ552811	KR132556	99 %	98.90 %	0 %	0.0
D-34B	*Micrococcus luteus*	OQ552812	KY486008	99 %	99.08 %	0 %	0.0
D-42A	*Bacillus cereus*	OQ552813	MH399242	99 %	97.73 %	0 %	0.0
D-23A	*Cytobacillus horneckiae*	OQ552814	MT397010	100 %	99.57 %	0 %	0.0
V-1C	*Vibrio owensii*	OQ552815	MN907475	99 %	99.17 %	0 %	0.0
V-2B	*Vibrio owensii*	OQ552816	CP045859	99 %	98.82 %	0 %	0.0
V-3A	*Vibrio owensii*	OQ552817	MZ148456	99 %	98.55 %	0 %	0.0
V-3B	*Vibrio owensii*	OQ552818	HQ908679	99 %	99.03 %	0 %	0.0
V-5A	*Vibrio harveyi*	OQ552819	MZ310506	99 %	99.03 %	0 %	0.0
V-6B	*Vibrio owensii*	OQ552820	MH093788	99 %	99.03 %	0 %	0.0

### 3.2 Construction and Analysis of Phylogenetic Tree

**Fig. 1:**
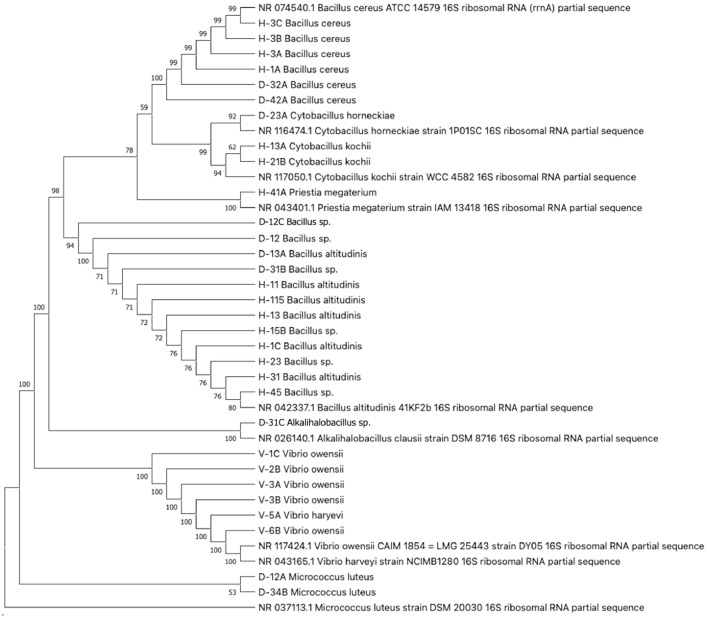
Phylogenetic tree of the endophytic bacteria from healthy and diseased *K. alvarezii* based on the 16S rDNA sequences.

Phylogenetic trees based on the 16S rDNA sequencing and other reference strains were constructed for all 31 isolates; 15 isolates from healthy *K. alvarezii* and 16 isolates from diseased *K. alvarezii*. In the phylogenetic tree of endophytes isolated from healthy *K. alvarezii*, the isolates were clustered into 3 major genera: *Bacillus, Cytobacillus*, and *Priestia*, as illustrated in Fig. 1. The isolates H-45, H-31, H-23, H-1C, H15B, H-13, H-11, and H-115 were found to be in the same branch as *Bacillus altitudinis*, with sequence similarities ranging from 99.24 % to 100 %. Meanwhile, the isolates H-13A and H-21B were clustered with *Cytobacillus kochii* with 99.17 % to 99.24 % similarities. The isolates H-3A, H-1A, H-3B and H-3C were clustered with *Bacillus cereus* with 98.19 % to 99.52 % similarities. Furthermore, the phylogenetic tree analysis of diseased *K. alvarezii* demonstrated 4 groups of endophytic bacteria: *Bacillus, Alkalihalobacillus, Vibrio* and *Micrococcus*. The isolates D-32A, D42-A, D-23A, D-12C, D-12, D-13A, and D-31B were clustered with *Bacillus altitudinis* and *Bacillus* sp. with 95.39 % to 100 % similarities. In addition, isolate D-31C was grouped with *Alkalihalobacillus sp.* with 83.02 % similarity. Moreover, the isolates V-1C, V-2B, V-3A, V-3B, V-5A and V-6B were placed in the same group as *Vibrio harveyi* and *V. owensii* with 98.55 % to 99.17 % similarities. The isolates D-34B and D-12A were grouped with *Micrococcus luteus* with 98.85 % to 99.08 % similarities.

## 4 Experimental Design, Materials, and Methods

### 4.1 Isolation of Endophytes from Healthy and Diseased *K. alvarezii*

The farmed seaweed was collected from Kampung Baru-Baru, Kota Belud (6.30228, 116.29455) and around Bum-Bum Island, Semporna (4.44747, 118.68691) in Sabah, Malaysia. The healthy seaweed samples were maintained in 35 ‰ (ppt) artificial seawater (NaCl 450 mM, KCl 10 mM, CaCl2 10 mM, MgCl2·6H2O 30 mM, MgSO4 30 mM, NaHCO3 2 mM) at temperature-controlled lab conditions, with temperature 23 °C ± 0.6 °C and pH range of 8.2–8.7 for optimum growth. The infected *K. alvarezii* samples were collected and stored separately from the healthy samples. All collected healthy seaweeds were washed in running water, and those with visible superficial injuries were excluded. The disinfection and isolation procedures were as follows: 70 % alcohol sterile, distilled water. The disinfection protocol was confirmed by plating the sterile water used to rinse the final wash in the TSA plate at 37 °C for 10 days. The absence of a microorganismal growth colony confirmed seaweed sterility [1]. Then, the surface-sterilized seaweed samples were homogenized using a sterile mortar and pestle. The tissue extract was subsequently incubated at 28 °C for 3 h to allow the complete release of endophytic microorganisms from the host tissue. For the isolation of endophytic bacteria, the tissue extracts were diluted with sterilized artificial seawater and plated on Tryptic Soy Agar (TSA), Bacto Agar (BA), Plant Agar (PA), and Marine Agar (MA) plates with different dilutions (10^−1^ and 10^−2^) and the plates were incubated for up to 15 days at 28 °C. On days 2, 5, 10, and 15, colonies were selected and purified using liquid broth. Endophytic bacterial colonies were chosen for each petri dish under consideration based on their stage of growth and morphology [2].

### 4.2 DNA Extraction

Wizard Genomic DNA Purification Kit (PROMEGA, USA) was used to extract the bacterial DNA. DNA extraction was performed according to the manufacturer's protocol [3]. 1 ml of overnight culture was centrifuged for 2 min at 13,000 x *g* to obtain pellet cells. The pelletized cells were lysed by adding 600 µl of Nuclei Lysis Solution, then incubated for 5 min at 80 ℃ followed by 3 µl of RNase solution and incubated at 37 ℃ for 30 min, the mixture was cool to room temperature. An additional step before the cells were lysed was added to colonies that might be gram-positive bacteria. For protein precipitation, 200 µl of Protein Precipitation Solution was added to the mixture. The mixture was vortexed, incubated on ice for 5 min and centrifuged at 13,000 x *g* for 3 min. After that, the supernatant was transferred to a clean microcentrifuge tube containing 600 µl isopropanol at room temperature. The mixture was gently mixed until thread-like strands of DNA were visible. The tube was centrifuged to obtain pellet cells and the supernatant was discarded. An amount of 600 µl of 70 % ethanol was added to the microcentrifuge containing pellet cells and centrifuged for 2 min at 13,000 x *g* and 70 % of ethanol was discarded. For the rehydration step, 50 µl of Rehydration Solution was added to the microcentrifuge tube. The microcentrifuge tube was centrifuged for 10 s at 13,000 x *g* and incubated at 65 ℃ then stored at 4 ℃. The verification of purity and expected bands were performed by Nanodrop and electrophoresis.

### 4.3 16s rRNA Gene PCR Amplification

16S rRNA gene PCR was performed using Velocity DNA Polymerase (Bioline, GERMANY), where the amplification was based on the standard manufacturer's protocol. Each reaction contained 10 µl of 5x Hi-Fi Reaction Buffer, 1.5 µl of DNA template, 1 µl of forward primer, 1 µl of reverse primer, 0.5 µl dNTPs mix, 1.5 µl of DMSO, 1 µl of DNA polymerase, 1 µl of MgCl_2_ and 32.5 µl of double-distilled water. The universal primers used to amplify the *16S* rRNA gene were 27 F (5”-AGAGTTTGATCMTGGCTCAG- 3”) and 1492 R (5”-GGTTACCTTGTTACGACTT-3”) [4]. The PCR amplification was performed in a thermal cycler machine (BioRAD PTC 200, USA) under standard cycling conditions. The PCR was performed at 95 ℃ for 1 min, 35 cycles, with each cycle consisting of 95 ℃ for 30 s, 65 ℃ for 30 s, 72 ℃ for 30 s, and finally 72 ℃ for 10 min [5]. The PCR products were stored at -20 ℃. Then, 2 µl of PCR product was examined by electrophoresis on 1 % agarose gel in TAE buffer. Then, the generated PCR products were cut and sent to Apical Scientific Snd Bhd (First Base Laboratories) for further PCR purification and DNA sequencing.

### 4.4 16S rDNA Sequencing and Analysis

The 16S rDNA sequences were edited by trimming low-quality regions, then the forward and reverse sequences of 16s rDNA were assembled using BioEdit (version 7.2) downloaded from (https://bioedit.software.informer.com/7.2/). DNA sequence homology searches were performed against sequences maintained in the NCBI GenBank database using a Blastn algorithm (http://www.ncbi.nlm.nih.gov/blast/Blast.cgi) [6]. The phylogenetic analysis and evolutionary distances were performed by applying the neighbour-joining method with bootstrap values of 1000 replicates in MEGA11 (http://www.megasoftware.net/index.html). MUSCLE in MEGA11 was used to align the edited sequences with their nearest matches to construct a phylogenetic tree, then the edited sequences were deposited in the GenBank under the accession numbers OQ552790 to OQ552820 [7].

## Ethics Statements

Not related.

## CRediT authorship contribution statement

**Vernon Vest Mangun:** Conceptualization, Methodology, Software, Formal analysis, Data curation, Visualization, Writing – original draft. **Rajeena Sugumaran:** Investigation, Data curation. **Wilson Thau Lym Yong:** Writing – review & editing, Supervision. **Nur Athirah Yusof:** Conceptualization, Methodology, Resources, Writing – review & editing, Supervision, Project administration, Funding acquisition.

## Data Availability

Dataset of 16S ribosomal DNA sequence-based identification of endophytic bacteria isolated from healthy and diseased Sabah red algae, Kappaphycus alvarezii (Original data) (NCBI Genbank).
